# Recent advances in copper-catalyzed asymmetric coupling reactions

**DOI:** 10.3762/bjoc.11.280

**Published:** 2015-12-15

**Authors:** Fengtao Zhou, Qian Cai

**Affiliations:** 1 Guangzhou Institutes of Biomedicine and Health, Chinese Academy of Sciences, No. 190 Kaiyuan Avenue, Guangzhou Science Park, Guangzhou 510530, P. R. of China; 2 Molecular Catalyst Research Center, Chubu University, Aichi, 487-8501, Japan

**Keywords:** asymmetric, carbon–heteroatom bond, copper, coupling

## Abstract

Copper-catalyzed (or -mediated) asymmetric coupling reactions have received significant attention over the past few years. Especially the coupling reactions of aryl or alkyl halides with nucleophiles became a very powerful tool for the formation of C–C, C–N, C–O and other carbon–heteroatom bonds as well as for the construction of heteroatom-containing ring systems. This review summarizes the recent progress in copper-catalyzed asymmetric coupling reactions for the formation of C–C and carbon–heteroatom bonds.

## Introduction

Copper-mediated coupling reactions, including the Ullmann [1], Ullmann–Goldberg [2–3], Ullmann diaryl ether formation [4] and Ullmann–Hurtley condensation [5], have been reported several decades before Pd and Ni-catalyzed reactions. However, the application of these methods was limited due to their disadvantages such as the requirement of stoichiometric amounts of copper and harsh reaction conditions (high temperatures). The turn of the millennium brought about the revival of the research in this field that was initiated by the use of soluble copper salts and ligand-coordinated Cu complexes as catalysts. This allowed the reactions to be carried out under much milder conditions. In the meantime these reactions have become one of the most classic, efficient and powerful methods for the formation of C–C, C–N, C–O and other carbon–heteroatom bonds. Extensive applications have been developed in both academia and industry [6–13]. Despite the progress in recent years, the research on asymmetric coupling reactions is still relatively rare. In this review, we highlight the developments in copper-catalyzed asymmetric coupling reactions, including the asymmetric coupling of aryl halides with nucleophiles for the formation of carbon–carbon and carbon–heteroatom bonds as well as the asymmetric allylic substitution with a wide range of nucleophiles for the formation of C–C and carbon–heteroatom bonds.

## Review

### Copper-catalyzed coupling of aryl halides with nucleophiles

#### Chiral auxiliary-induced aryl C–C coupling

**Scheme 1:**
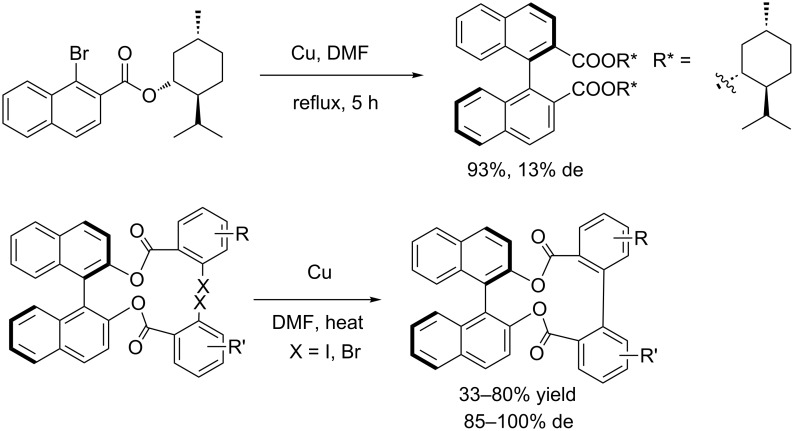
Copper-catalyzed asymmetric preparation of biaryl diacids by Ullmann coupling.

The biaryl motif is a key subunit in many natural products and axially chiral ligands. The classical Ullmann coupling is one of the most important methods for the practical synthesis of biaryls [14]. However, only few reports of an asymmetric version of the Ullmann coupling have been documented. The first attempt of an intermolecular asymmetric Ullmann coupling for the formation of biaryls was reported by Miyano and co-workers in 1980. The authors used a chiral ester group as auxiliary but only poor diastereoselectivity (13% de) was obtained [15]. A few years later, the same group took advantage of a chiral (*R*)-BINOL-bridge to link the two aromatic acids and obtained the coupling product with excellent stereocontrol (up to 100% de) (Scheme 1) [16–19].

**Scheme 2:**
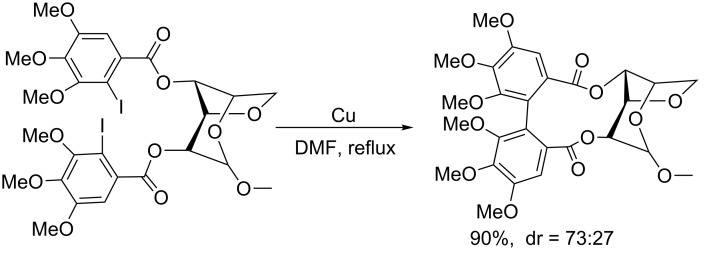
Intramolecular biaryl coupling of bis(iodotrimethoxybenzoyl)hexopyranose derivatives.

In 1998, Martin et al. [20] applied this strategy to the asymmetric intramolecular biaryl coupling of sugar derivatives carrying 2-iodo-3,4,5-trimethoxybenzoyl substituents (Scheme 2).

**Scheme 3:**
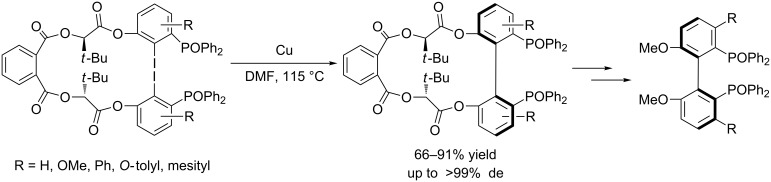
Preparation of 3,3’-disubstituted MeO-BIPHEP derivatives.

In 2006, Keay et al. [21] successfully developed an intramolecular asymmetric Ullmann coupling for the preparation of 3,3’-disubsituted MeO-BIPHEP derivatives using a chiral ester auxiliary that was easily prepared from (*R*)-2-hydroxy-3,3-dimethylbutyrate. In this reaction, only one diastereoisomer was formed during the Ullmann coupling and the auxiliary is easily prepared and removed by hydrolysis after the coupling reaction (Scheme 3).

**Scheme 4:**
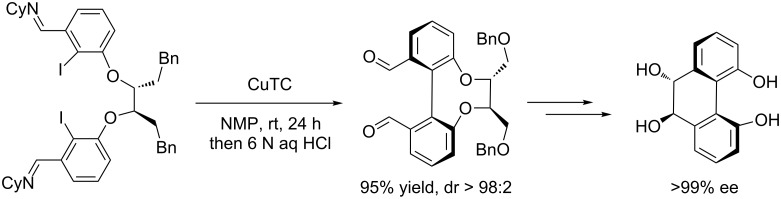
Enantioselective synthesis of *trans*-4,5,9,10-tetrahydroxy-9,10-dihydrophenanthrene.

In 2007, Breit et al. [22] employed a chiral tether to link two aryl halides for the enantioselective synthesis of *trans*-4,5,9,10-tetrahydroxy-9,10-dihydrophenanthrene at room temperature (Scheme 4).

In 1994, Meyers and Nelson [23–24] developed a copper-mediated asymmetric biaryl coupling with oxazoline as the chiral auxiliary to afford the biaryl-coupling products in high diastereomeric purity (dr = 93:7, Scheme 5).

**Scheme 5:**
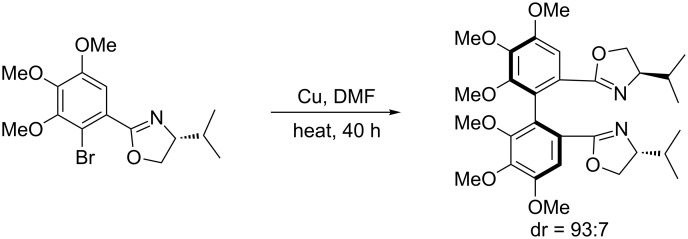
Copper-catalyzed coupling of oxazoline-substituted aromatics to afford biaryl products with high diastereomeric purity.

**Scheme 6:**
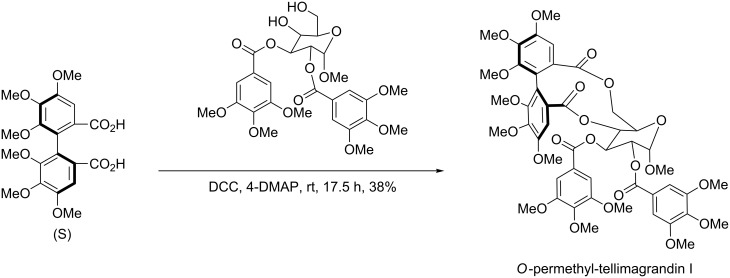
Total synthesis of *O*-permethyl-tellimagrandin I.

**Scheme 7:**
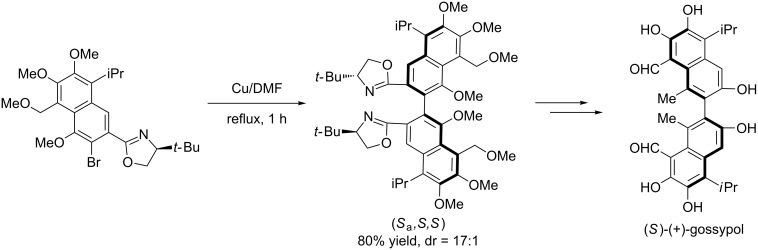
Total synthesis of (+)-gossypol.

**Scheme 8:**
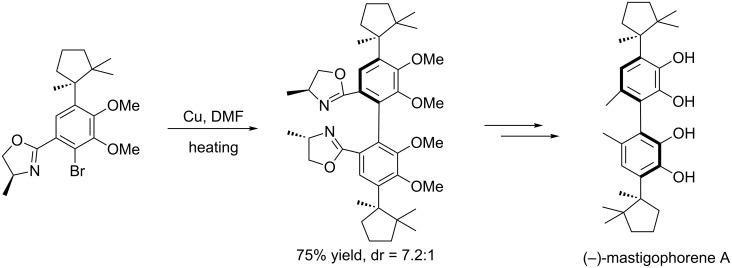
Total synthesis of (−)-mastigophorene A.

Meyers et al. also successfully applied the aforementioned strategy to the asymmetrical synthesis of many natural products [25–27] such as *O*-permethyl-tellimagrandin I (Scheme 6), (+)-gossypol (Scheme 7), (−)-mastigophorene A (Scheme 8).

**Scheme 9:**
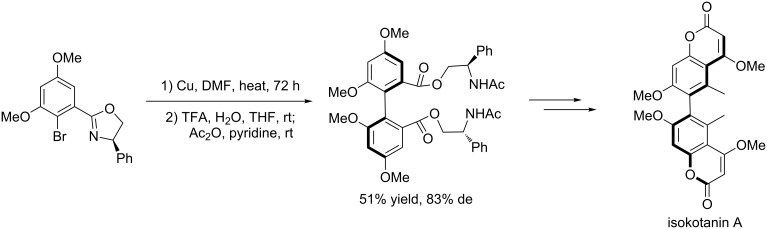
Total synthesis of isokotanin.

**Scheme 10:**
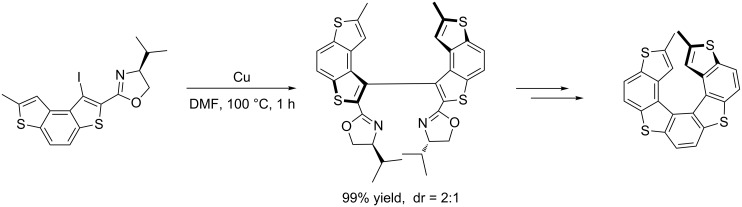
Synthesis of dimethyl[7]thiaheterohelicenes.

Based on this strategy, Lin and Zhong [28] developed an efficient method for the synthesis of Isokotanin A (Scheme 9) and Tanaka et al. [29] also used this method for the synthesis of dimethylthiaheterohelicenes, which are highly hindered *C*_2_-symmetrical biaryls (Scheme 10).

**Scheme 11:**
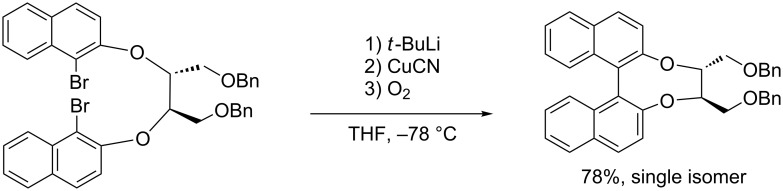
Intramolecular coupling with chiral ortho-substituents.

In 1994, an impressive progress was made by Lipshutz [30] in the intramolecular oxidative biaryl-coupling through the formation of higher-order cyanocuprates. The authors realized an asymmetrical intramolecular reaction by means of inexpensive optically active auxiliary bridges. The most efficient chiral auxiliary was found to be a *C*_2_-symmetrical bridge bearing two stereogenic centers, derived from tartaric acid, giving the product as a single isomer in good yield (Scheme 11).

**Scheme 12:**
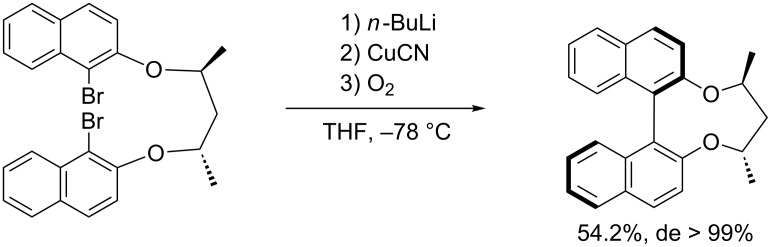
Chiral 1,3-diol-derived tethers in the diastereoselective synthesis of biaryl compounds.

Sugimura et al. [31] expanded this method by introducing chiral 1,3-diol-derived tethers into the substrates, delivering the corresponding coupling products in excellent diastereoselectivity (Scheme 12).

**Scheme 13:**
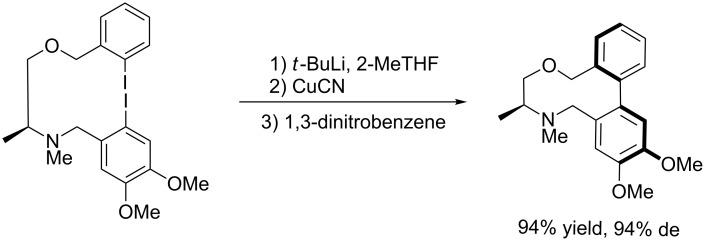
Synthesis of chiral unsymmetrically substituted biaryl compounds.

Schreiber et al. [32] reported an efficient preparation of axially chiral unsymmetrical biaryl compounds in good to excellent diastereoselectivities by coupling through the formation of higher-order cuprates (Scheme 13).

**Scheme 14:**
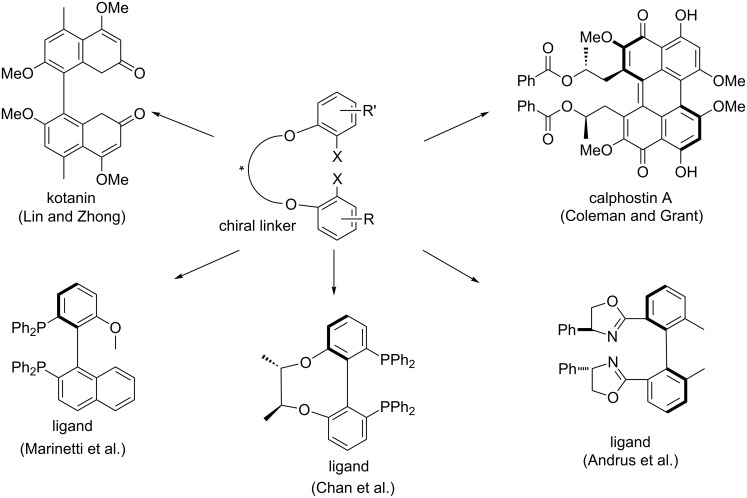
Atroposelective synthesis of biaryl ligands and natural products by using a chiral diether linker.

The utility of this strategy was also demonstrated by the atroposelective synthesis of many very useful axially chiral ligands and biologically important natural products. Some representative examples of these compounds are collected in Scheme 14. In 1997, Andrus et al. [33] used this method for the synthesis of enantiomerically pure bisoxazoline. Lin and Zhong [34] synthesized the natural product kotanin. Coleman and Grant [35] described an efficient synthesis of calphostin A, a potent protein kinase C inhibitor. In 2002, Marinetti et al. [36–37] employed this approach to prepare biaryl diphosphines. In 2004, Chan et al. [38] also developed a diastereoselective synthesis of chiral biphenyl diphosphine ligands by means of an intramolecular Ullmann coupling with the introduction of chiral bridged ethers.

#### Catalytic asymmetric C–C coupling

**Scheme 15:**
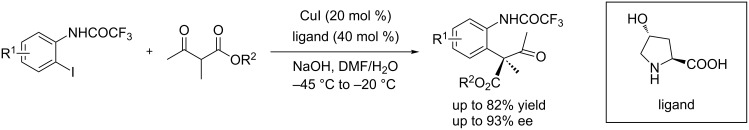
Enantioselective arylation reactions of 2-methylacetoacetates.

In 1929, Hurtley reported the first example of a C-arylation reaction of malonic esters with 2-bromobenzoic acid using a catalytic amount of copper-bronze or copper acetate [5]. Later on, great progress has been made in this reaction, allowing it to be carried out under practically useful and mild conditions [9–11]. However, an enantioselective version of this type of reaction remained challenging. Up to 2006 the report by Ma et al. [39] has been the only example of this type of catalytic asymmetric coupling reaction. They reacted 2-halotrifluoroacetanilides with 2-methylacetoacetates under the catalysis of CuI/*trans*-4-hydroxy-L-proline and obtained the arylated products in good yields and enantioselectivities. In this reaction, the trifluoroacetamido moiety present in the ortho position of the aryl halides plays an important role in enantiocontrol (Scheme 15).

#### Copper-catalyzed asymmetric aryl C–N coupling through desymmetrization and kinetic resolution strategies

In the past, the asymmetric version of aryl C–N/O/S coupling reactions has not attracted the attention from the organic chemistry community. This may have been due to the fact that these reactions do not allow for the direct creation of new stereochemical centers. Only a few examples were reported for asymmetric N-arylation reactions using a Pd catalytic system through an “indirect” way, either by asymmetric desymmetrization or kinetic resolution [40–44]. In most cases, the enantioselectivities were not satisfactory. Recently, a copper catalytic system became another option toward asymmetric N-arylation reactions in term of improving enantioselectivity and efficiency.

**Scheme 16:**
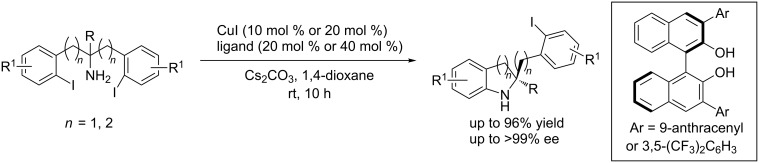
Asymmetric aryl C–N coupling reactions following a desymmetrization strategy.

In 2012, Cai et al. [45] developed the first copper-catalyzed asymmetric intramolecular Ullmann C–N coupling reaction through a desymmetrization strategy. The reaction lead to the enantioselective formation of indolines and tetrahydroquinolines in high yields and up to >99% ee (Scheme 16).

**Scheme 17:**
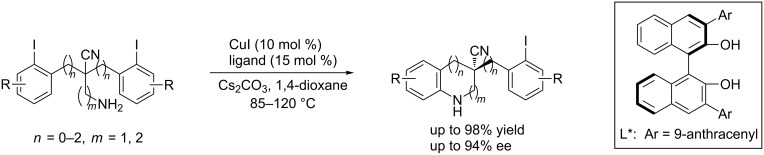
Construction of cyano-bearing all-carbon quaternary stereocenters.

In 2014, Cai et al. [46] applied the desymmetrization strategy to construct chiral cyano-bearing all-carbon quaternary stereocenters, affording 1,2,3,4-tetrahydroquinoline analogues in good yields and excellent enantioselectivities (Scheme 17).

**Scheme 18:**
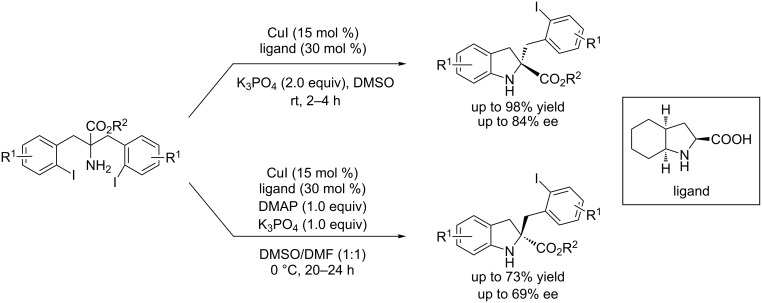
An unexpected inversion of the enantioselectivity in the asymmetric C–N coupling reactions using chiral octahydro-1*H*-indole-2-carboxylic acid as the ligand.

The same group also observed that achiral additives such as 4-(*N*,*N*-dimethylamino)pyridine caused an unexpected inversion of enantioselectivity in the Cu-catalyzed asymmetric desymmetrization of α,α-bis(2-iodobenzyl)glycines when (2*S*,3a*S*,7a*S*)-octahydro-1*H*­indole-2-carboxylic acid was used as chiral ligand [47] (Scheme 18).

**Scheme 19:**
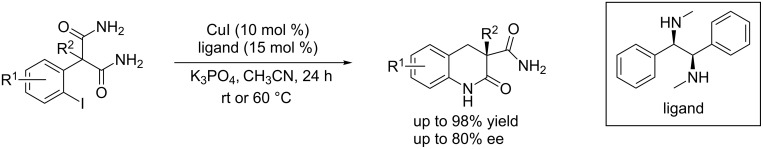
Differentiation of two nucleophilic amide groups.

In 2015, Cai et al. reported another type of desymmetrization process, which allowed for the discrimination between two symmetric nucleophilic amine-type groups. Enantiocontrol using such substrates is more difficult in the asymmetric desymmetric aryl C–N coupling reaction because the two nucleophilic groups may serve as good chelating ligands and thus compete with the chiral ligand for binding with the copper salts. Therefore the authors used a mono-aryl halide-substituted malonamide in the presence of a chiral CuI/1,2-diamine catalyst system and obtained the desired products in good yields and moderate enantioselectivities [48] (Scheme 19).

**Scheme 20:**
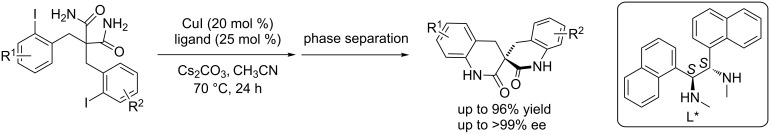
Synthesis of spirobilactams through a double N-arylation reaction.

This method was further applied to a double N-arylation reaction for the enantioselective formation of spirobilactams by Cai et al. [49]. Through the combination of the copper-catalyzed double N-arylation and a simple in situ solid–solution phase separation, the spirobilatams were formed in good yields and with excellent enantioselectivities (Scheme 20).

**Scheme 21:**
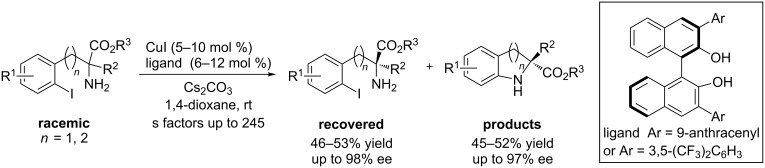
Asymmetric N-arylation through kinetic resolution.

Kinetic resolution is another strategy for asymmetric aryl C–N coupling reactions. Cai et al. [50] developed a copper-catalyzed asymmetric intramolecular N-arylation of *rac*-2-amino-3-(2-iodoaryl)propionates and *rac*-2-amino-4-(2-iodoaryl)butanoates with CuI/BINOL-derived ligands in 2013, affording the chiral coupling products and recovered starting material with high enantioselectivity (Scheme 21).

**Scheme 22:**
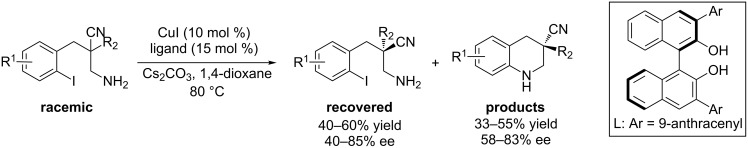
Formation of cyano-substituted quaternary stereocenters through kinetic resolution.

Recently, the aforementioned kinetic resolution strategy was applied to another type of substrates [51], leading to the formation of cyano-substituted quaternary stereocenters (Scheme 22).

#### Asymmetric C–O coupling

**Scheme 23:**
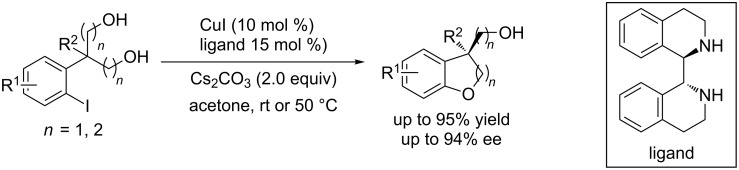
Copper-catalyzed intramolecular desymmetric aryl C–O coupling.

Numerous methods have been developed during the last two decades for the formation of aryl C–O bonds but asymmetric aryl C–O coupling is still a challenge [6–10]. In 2013, Beaudry and Quamar Salih reported the first copper-catalyzed asymmetric diaryl ether formation in the synthesis of (−)-myricatomentogenin, (−)-jugcathanin, (+)-galeon and (+)-pterocarine [52]. However, the enantioselectivity was poor in most cases. In 2013, Cai and co-workers [53] reported the first Pd-catalyzed highly enantioselective intermolecular aryl C–O coupling reaction for the construction of chiral (3,4-dihydro-2*H*-chromen-3-yl)methanols in good yields and high enantioselectivity by means of a desymmetrization strategy. A modified palladium catalytic system with a SDP(O) ligand was developed in 2015 for the asymmetric desymmetrization of 2-(2-halophenoxyl)-1,3-diols by the same group [54]. However, the palladium catalytic systems suffered from limited substrate scope and poor efficiency and enantioselectivity for the formation of quaternary stereocenters. Recently, Cai et al. carried out such couplings using a CuI/cyclized diamine catalytic system for the formation of 2,3-dihydrobenzofurans and analogs [55]. The copper catalytic system proved very efficient and compatible with a wide range of substrates under mild conditions. It overcame the shortcomings of the palladium catalytic system for the formation of quaternary stereocenters (Scheme 23).

### Copper-catalyzed couplings of allylic halides with nucleophiles

**Scheme 24:**
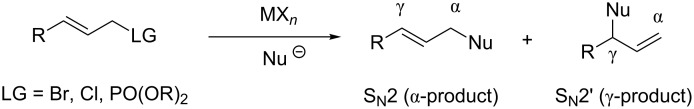
Transition metal-catalyzed allylic substitutions.

Transition metal-catalyzed allylic substitutions are the most important process for carbon–carbon and carbon–heteroatom bond formation in organic synthesis [56–58]. Allylic substitution of the substrate with nucleophiles can afford two different products: the S_N_2-product or the S_N_2’-product (Scheme 24).

Usually, S_N_2’ regioselective allylic substitutions, which create a new stereogenic center, are more valuable. Methods that allow the S_N_2’ regioselective C–C bond formation have been extensively studied over the past years. In contrast to other metals (Pd, Mo, and Ir), copper-catalyzed allylic substitution reactions allow the use of nonstabilized nucleophiles including organomagnesium, organoaluminum, organozinc and organoborane reagents. Moreover, copper-catalyzed allylic substitution reactions usually proceed with high S_N_2’ regioselectivity, creating a new stereogenic center [59–60].

In 1995, Bäckvall et al. reported the first example of an asymmetric allylic substitution reaction catalyzed by a chiral copper complex, giving a moderate enantioselectivity (42% ee) in Grignard reactions with allylic acetates. The enantiomeric excess was later improved to 64% by using a new chiral ferrocenyl ligand [61–62]. Subsequently, great progress has been made in the development of copper-catalyzed asymmetric allylic substitution reactions. These considerable progresses have been reviewed by Hoveyda [56], Oshima [57], Alexakis [58], Feringa [59] and Diéguez [60]. In this review, we focus on the developments since 2008.

#### Cu-catalyzed enantioselective allylic substitutions with aryl-, alkenyl-, and allenylboronates, alkylboron compounds

**Scheme 25:**
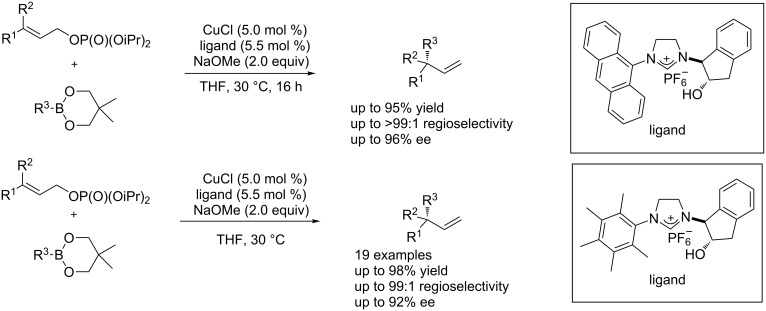
Copper-catalyzed asymmetric allylic substitution of allyl phosphates.

Organoboron compounds have found extensive application in coupling reactions for the construction of C–C bonds [63]. Recently, the copper-catalyzed enantioselective allylic substitutions with organoboron compounds have seen impressive progress. In 2011, Hayashi et al. [64] developed a new efficient method for the highly regioselective and enantioselective construction of tertiary carbon stereocenters by the copper/NHC-catalyzed asymmetric allylic substitution of allyl phosphates with arylboronates. Furthermore, they applied the method to the construction of quaternary carbon stereocenters with good enantioselectivity (up to 90% ee) with disubstituted allyl phosphates. The enantioselectivity was later improved to 92% ee with a new chiral catalyst (Scheme 25) [65].

**Scheme 26:**
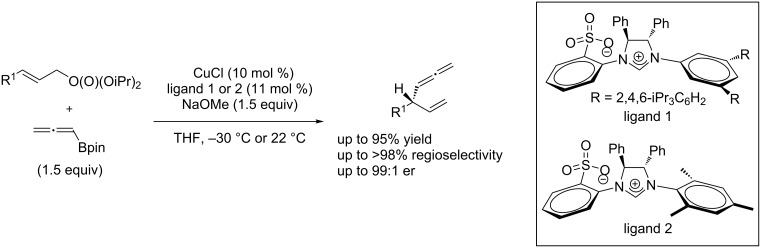
Allylic substitution of allyl phosphates with allenylboronates.

In 2012, Hoveyda and Jung reported a copper/NHC-catalyzed asymmetric allylic substitution of allyl phosphates with allenylboronates [66], leading to chiral allenes bearing a tertiary or quaternary carbon stereogenic center in high yields and with excellent enantioselectivity (Scheme 26).

**Scheme 27:**
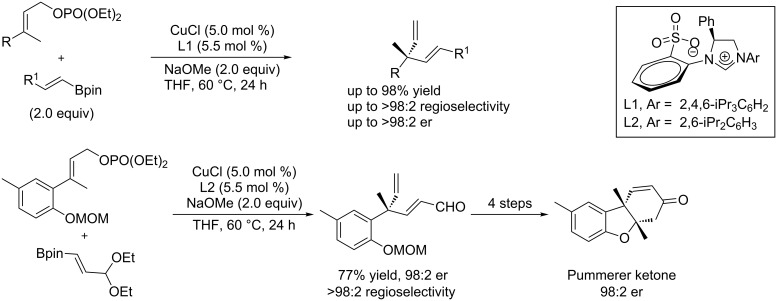
Allylic substitution of allyl phosphates with vinylboron.

The copper/NHC catalyst system was also applied to the allylic substitution of allyl phosphates with commercially available or easily accessible vinylboron reagents, leading to chiral alkenes bearing a quaternary carbon stereocenter. The utility of this protocol was demonstrated by the concise enantioselective syntheses of the Pummerer ketone (Scheme 27) [67].

**Scheme 28:**
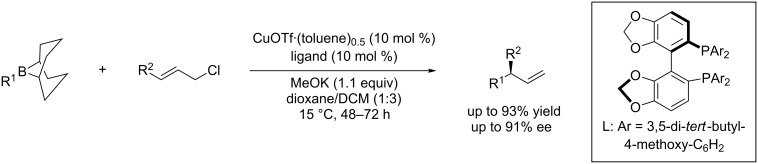
Allylic substitution of allyl phosphates with vinylboron.

In 2012, Sawamura et al. reported a Cu(I)-DTBM-SEGPHOS-catalyzed enantioselective allylic substitution reaction with alkylboron compounds [68]. In this report, alkyl-9-BBN reagents for the first time served as nucleophiles reacting with primary allylic chlorides with excellent γ-selectivity and with high enantioselectivity (Scheme 28).

**Scheme 29:**
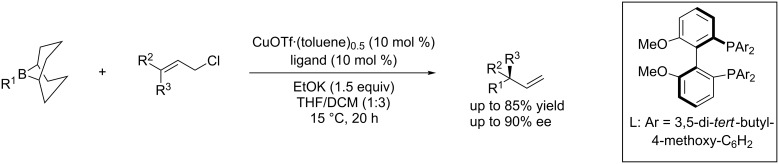
Construction of quaternary stereogenic carbon centers through enantioselective allylic cross-coupling.

This catalytic system is however restricted to the construction of tertiary carbon stereocenters. An improved catalytic system [69–70] allowed disubstituted primary allyl chlorides to react with alkylborane (alkyl-9-BBN) for the generation of a quaternary carbon stereogenic center bearing three sp^3^-alkyl groups and a vinyl group with an ee up to 90% (Scheme 29).

#### Cu-catalyzed enantioselective allylic substitutions with Grignard reagents

**Scheme 30:**
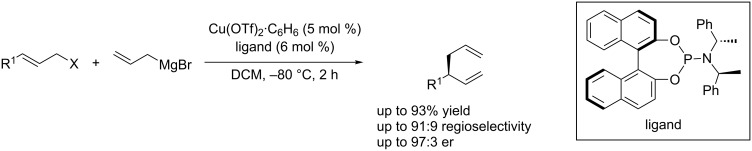
Cu-catalyzed enantioselective allyl–allyl cross-coupling.

Transition metal-catalyzed allyl–allyl cross-coupling of allylmetal species with allylic electrophiles represents one of the powerful methods to establish 1,5-dienes. These compounds are abundant in natural terpenes as well as highly versatile intermediates in organic synthesis [71–73]. However, highly enantioselective allyl–allyl cross-coupling was only achieved by employing a Pd-catalyst system [74]. Copper-catalyzed asymmetric allylic alkylation (Cu-AAA) with allylmetal is still a great challenge. In 2013, Feringa et al. [75] reported the first copper-catalyzed highly enantioselective allyl–allyl cross-coupling of allyl Grignard reagents with allyl bromides, leading to chiral 1,5-dienes in good yield and with high enantioselectivity (Scheme 30).

#### Cu-catalyzed enantioselective allylic substitutions with silylboronates

**Scheme 31:**
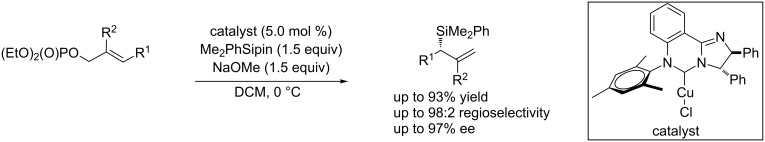
Cu-catalyzed enantioselective allylic substitutions with silylboronates.

Enantioenriched allylsilanes are very useful building blocks in synthetic organic chemistry [76]. Oestreich et al. [77] reported the first example of an enantio- and regioselective allylic substitution of linear allylic chlorides and phosphates catalyzed by a Cu/NHC chiral ligand (Scheme 31).

**Scheme 32:**
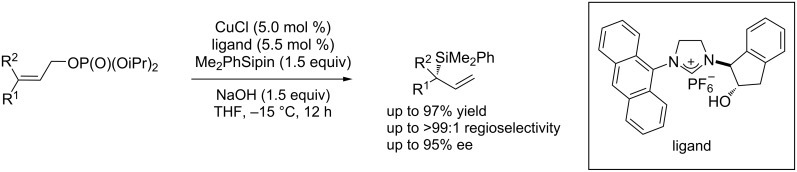
Asymmetric allylic substitution of allyl phosphates with silylboronates.

Hayashi et al. [78] described a Cu/NHC-catalyzed asymmetric allylic substitution of allyl phosphates with silylboronates that provides a straightforward access to chiral allylsilanes with high regio- and enantioselectivity (Scheme 32).

#### Cu-catalyzed enantioselective allylic substitutions with diboronates

**Scheme 33:**
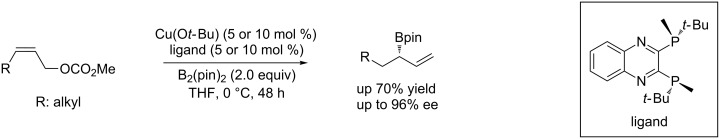
Stereoconvergent synthesis of chiral allylboronates.

Chiral allylboronates are useful reagents, which could be manipulated in a number of useful ways to give functionalized chiral building blocks such as allylic alcohols, amines etc. [79]. Sawamura et al. [80] developed a highly enantioselective copper-catalyzed asymmetric allylic substitution with diboronates to afford chiral allylboronates (Scheme 33).

**Scheme 34:**
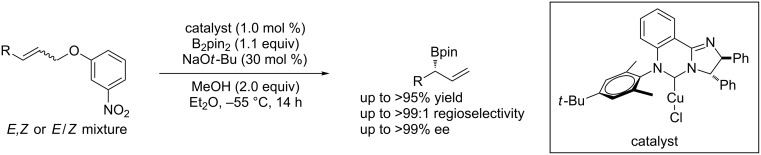
Enantioselective allylic substitutions with diboronates.

McQuade et al. [81] reported a copper/NHC-catalyzed allylic substitution of aryl ether substrates with diboron or α-substituted allylboronates in good yield and with high enantioselectivity. The reactions exhibited a wide functional-group tolerance with pure *E* or *Z* isomers or *E*/*Z* alkene mixtures (Scheme 34).

#### Cu-catalyzed enantioselective allylic substitutions with terminal alkynes

**Scheme 35:**
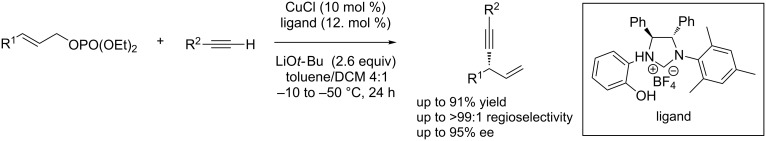
Enantioselective allylic alkylations of terminal alkynes.

The catalytic enantioselective allylic alkylation of alkynyl nucleophiles is a powerful tool for the preparation of 1,4-enynes, which are versatile synthetic intermediates in asymmetric organic synthesis [82]. In 2014, Sawamura et al. [83] successfully developed a highly enantioselective allylic alkylation of terminal alkynes with primary allylic phosphates through a copper/NHC chiral catalyst system. The authors obtained chiral enynes with a tertiary stereocenter at the allylic propargylic position in good yield and with excellent enantioselectivity (Scheme 35).

## Conclusion

Copper-catalyzed or -mediated enantioselective carbon–carbon or carbon–heteroatom coupling reactions have been one of the most challenging areas in asymmetric catalysis in recent years. The asymmetric copper-catalyzed C(_Aryl_)–C(_Aryl_) bond formation through the incorporation of a chiral ester group as auxiliary into the substrates, has emerged as a powerful tool for constructing natural products and useful ligands with axial chirality. Based on asymmetric desymmetrization and kinetic resolution strategies, a series of efficient copper-catalyzed systems have been developed for the formation of C–C, C–N, C–O bonds and the construction of nitrogen- and oxygen-containing ring systems. Despite the substantial progress that has been made in copper-catalyzed or -mediated enantioselective carbon–carbon and carbon–heteroatom coupling reactions, limitations in terms of substrate scope and catalyst efficiency still exist. In the future, the design of new ligands and improved catalyst systems are required that allow for transformations of more challenging substrates.
